# Geomimetics and Extreme Biomimetics Inspired by Hydrothermal Systems—What Can We Learn from Nature for Materials Synthesis?

**DOI:** 10.3390/biomimetics2020008

**Published:** 2017-05-31

**Authors:** Miriam M. Unterlass

**Affiliations:** Institute of Materials Chemistry, Technische Universität Wien, 1060 Vienna, Austria; miriam.unterlass@tuwien.ac.at; Tel.: +43-158-801-165206

**Keywords:** materials synthesis, extreme biomimetics, geomimetics, hydrothermal synthesis, hybrid materials, green materials chemistry

## Abstract

‘Extreme biomimetics’ and ‘geomimetics’ are relatively recent fields of materials chemistry. Both take inspiration from natural materials for generating novel synthetic materials or enhanced properties in known materials. In geomimetics, the source of inspiration is geological systems, while extreme biomimetics is motivated by organisms operating in—from an anthropocentric point of view—extreme conditions. This review article focuses on geomimetic and extreme biomimetic hydrothermal synthesis. Since hydrothermal preparative chemistry typically uses nothing but water and the required precursors, the field belongs to the research area of ‘green materials chemistry’. Geomimetics, on the one hand, takes inspiration from natural materials formation. Extreme Biomimetics, on the other hand, is inspired by materials found in extremophile organisms, instead of aiming to implement their actual biosynthesis. In this contribution, both extreme biomimetics and geomimetics are first defined, and further critically discussed on the basis of recent, selected examples. Moreover, the necessity for the two closely related fields as well their prospects are commented on.

## 1. Introduction

Materials are of utmost importance for complex societies: civilization is inevitably linked to technology, which is, in turn, enabled by the availability and mastery of materials. Indeed, whole eras are named after the predominant material of their time, such as the Bronze Age or the Iron Age. Nowadays, there is an ever-growing demand for more and more sophisticated and performant materials—a direct consequence of the augmenting complexity in modern technology. At the same time, mankind is increasingly aware of the environment and human health, as underlined e.g., by the ‘12 Principles of Green Chemistry’ [1]. Therefore, harmful materials synthesis and processing simply do not justify their means anymore. One possibility for developing novel and less harmful materials and routes towards such materials is by taking inspiration from natural systems, for instance by employing geomimetic and extreme biomimetic approaches. In fact, most interesting materials are generated in biological systems operating in extreme surroundings, as well as in non-living systems such as inorganic geological environments. Both are worth taking inspiration from.

Biomaterials are designed most efficiently, typically involving a hierarchical structure from the nano- to the macroscale. Such hierarchical structuring allows a wide range of outstanding properties to be obtained from a limited number of relatively simple building blocks. The archetypical example of the latter is provided by the shells of mollusks, also known as nacre. Nacre shows both high hardness and high fracture toughness, despite being made of the mechanically low-performing components ${\mathrm{CaCO}}_{3}$ and proteins [2]. This is achieved by combining ${\mathrm{CaCO}}_{3}$ nanoparticles and biopolymers into a nanostructured, hierarchical hybrid material [3]. The field of *biomimetics*, which has now been thriving for approximately 60 years [4], has always been driven by taking inspiration from biological structures (e.g., biominerals, biological surfaces), and by aiming to understand their formation and function. Until recently, the field was mainly concerned with biological systems that could be more or less easily discovered by humans and that operate at ambient conditions, such as the above mentioned mollusk shells, lotus leaves or morpho butterflies, for instance. In recent years, species that operate in classically inaccessible surroundings—such as extremophile microorganisms inhabiting hot acidic springs, or deep see fauna—have started to be heavily investigated. This has previously either not been possible due to technological limitations (deep sea organisms), or recently been driven by other disciplines (biotechnological interest in extremophile microorganisms). Such organisms represent the most interesting subjects of inspiration, which only approximately 10 years ago led to the birth of extreme biomimetics. In this field, a plethora of study subjects is conceivable, including an understanding, spanning across species, of the underpinnings of their resistance to extreme habitats, or an understanding of the formation process of biominerals in extremophiles. To date, the field has been mainly concerned with synthesizing materials that are inspired by biominerals in extremophiles—almost certainly a consequence of its youth. A major part of extreme biomimetic materials has to date been achieved by hydrothermal synthesis.

Geological, non-living environments are the birthplace of countless mostly inorganic and crystalline materials. Modern chemistry, physics and geosciences have been mimicking and aiming to understand natural crystallization processes, such as crystallization from solution, the melt or via vapor-transport reactions, for over 100 years. In the field of hydrothermal synthesis of materials, researchers have, to date, been mainly concerned with the preparation of inorganic compounds [5], or amorphous carbons [6,7]. In contrast to extreme biomimetic materials that are inspired by materials in extremophiles, geomimetics is rather inspired by natural, geological syntheses. Most recently, geomimetics has been broadened by an additional class of materials that can be obtained hydrothermally: crystalline organic materials [8]. There are important analogies in the hydrothermal synthesis of crystalline organic materials, and the natural hydrothermal crystallization of minerals. These analogies are rooted in highly similar mechanisms of the formation reactions of both, which are (poly-)condensation reactions. Therefore, the attribution of these materials and their syntheses as geomimetic are most appropriate.

Recent developments in extreme biomimetics and geomimetics materials are centered around hydrothermal approaches and therefore, synthesis-wise, have a lot in common. This review article is aimed at discussing the synthetic conditions, related structures and properties that are obtained at elevated temperatures and pressures in aqueous systems. For the use of water as the only reaction medium, hydrothermal techniques are intrinsically benign and are thus green routes towards materials. In the following, the conditions of extreme biomimetics and geomimetics will be briefly summarized, including a short description of organisms operating in such conditions and geological examples, respectively. Both common points and differences between extreme biomimetics and geomimetics will be discussed. Then, the physicochemical properties of water at high temperature (*T*), and also pressure (*p*), will be briefly reviewed. Furthermore, selected examples of materials syntheses mimicking such conditions will be discussed. In the last section, the benefits and drawbacks as well as future potential for extreme biomimetic and geomimetic approaches will be commented on. Overall, this review article is intended to serve as a starting point for materials scientists, chemists and physicists, as well as interested undergraduate and graduate students; this is ensured by a tutorial writing style.

Before starting the discussion, a few short remarks shall be made, regarding the benefits of looking into extreme biomimetics and geomimetics, for scientists and engineers in general. The term ‘mimicry’ originates from the ancient Greek μ*î*μoς (English: “imitator”, “actor”) and implies an actual inspiration by something else, leading to its imitation/copying. However, great scientific/technological developments often happen by accident rather than as an outcome of a meticulously planned strategy (or as a non-anticipated outcome of following a meticulously planned strategy). Hence, if a researcher were to synthesize a hybrid material composed of calcite nanocrystals and a biopolymer, would the resulting hybrid be biomimetic even if the researcher did not aim to imitate nacre? Intended mimicry or not, there is huge benefit in comparing a synthetic material to a naturally formed analogon, as material properties are strongly structure-related, and structures are strongly influenced by synthetic conditions. It is therefore advisable for materials chemists, physicists and engineers to be aware of extreme biomimetics and geomimetics. Moreover, the fact that fully organic crystalline materials can be generated hydrothermally, is surprising and counterintuitive. Learning why and how these syntheses are possible at such non-classical reaction conditions is therefore certainly also of interest to organic chemists.

## 2. Discussion

### 2.1. ‘Extreme’ Biological Conditions

‘Extreme’ ethymologically originates from the Latin *extremus* meaning “situated at the end, edge or tip”, which is the superlative of *exter*, meaning “outer, outward, on the outside”. Hence, ‘extreme’ refers to a value at the very end of a scale, where a certain frame of reference sets the limits of that scale. In materials synthesis, such scales are typically the physicochemical conditions in which that synthesis takes place, i.e., parameters such as *T* or *p*. If the values that the latter parameters occupy are extreme or not depends on the frame of reference: a given *T* can be ‘extreme’ in one case, and hugely ‘benign’ in another scenario. Consider for instance the ‘sol–gel process’, which is an outstandingly clever procedure for generating ceramics (mostly metal oxides) from soluble and hence processable metal alkoxide (also known as alkoholate) species [3,9]. The classical temperature range for generating such a gel is around 25–100 ${}^{\circ }$C [10]. Compared to more classical manufacturing and processing conditions for ceramics (such as sintering techniques; several hundred to >1000 ${}^{\circ }$C), sol–gel processes are surprisingly benign. Therefore, they are often referred to as a *chimie douce* (French for ‘soft chemistry’) approach [11]. However, if one thinks about a typical sol–gel process temperature of say 80 ${}^{\circ }$C in the context of a human cell, these conditions would be detrimental, even disastrous for that cell. In other words: extreme.

#### 2.1.1. Extremophile Microorganisms

The term *extreme biomimetics* implies that there are biological systems working at the extreme end of conventional conditions for living systems. In fact, there is a number of organisms living in extreme environments, which are summarized under the term extremophiles. According to the definition, extremophiles thrive best under conditions that are harsh as compared to conventional environments. Note that in contrast to extremophiles, extremotolerant microorganisms do not necessarily thrive best under extreme conditions, but can survive. Microorganisms belonging to the subcategory of obligate extremophiles are bound to the extreme conditions that they favor for survival. Research on extremophiles has been flourishing in recent years, for—amongst other reasons—(i) the connection to the ‘Origin of Life’, i.e., the first form of life on earth had to deal with much harsher conditions than those faced by most current organisms [12]; (ii) the importance to astrobiology, e.g., concerning the definition of ‘habitable conditions’ of interest in the search for extraterrestrial life as well as the transport of life from earth to another planet [13,14]; and (iii) the importance to biotechnology, where e.g., enzymes from extremophiles can perform biocatalysis in higher *T* or much more acidic conditions than those in which conventional enzymes would subsist [15].

**Figure 1:**
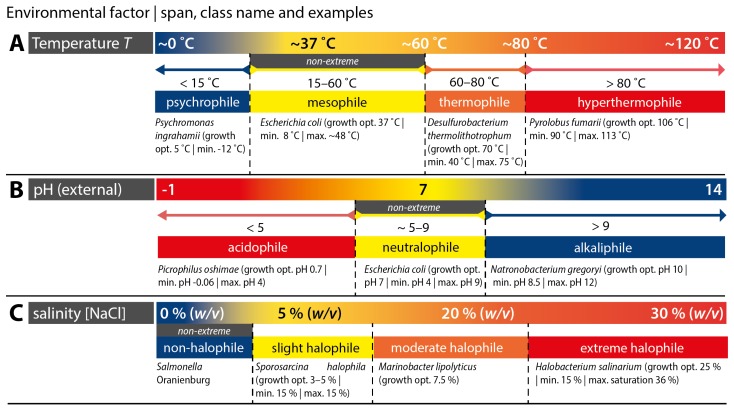
Classes of extremophiles with respect to temperature (*T*), pH and [NaCl]. Subdivision of microorganisms favoring different temperatures (**A**), external pH values (**B**), and salinity (**C**) are given, including the name of the class, and examples with minimal, optimal and maximal growth with respect to the parameter. Compiled using data from references [12,13,16,17,18,19,20,21,22,23,24,25].

Extremophiles are found in all three domains of life, i.e., in Archaea, Bacteria and Eukarya [12]. An overview of extremophiles for three exemplary environmental factors (*T*, pH and concentration of sodium chloride [NaCl]) is depicted in Figure 1. The classification of extremophiles is commonly done according to the physicochemical parameter at which they operate, when the value of the parameter is extreme as compared to—from an anthropocentric point of view—‘normal’ conditions.

Classified according to *T* of the environment, humans or well-known examples in the microbial world such as *Escherichia coli* belong, temperature-wise, to the class of moderate thermophiles also known as mesophiles [18]. However, there are organisms that thrive at lower temperatures, which are found in habitats such as permafrost (e.g., found in the Arctic or Antarctic), or in the deep sea. *Psychromonas ingrahamii* is such a so-called psychrophile, i.e., a cold-loving extremophile, which grows best at 5 ${}^{\circ }$C, but can still grow down to –12 ${}^{\circ }$C [12]. In hotter than ‘normal’ habitats, such as hot springs or the surroundings of submarine hydrothermal (HT) vents (for instance black smokers) one finds organisms that prefer higher *T*, i.e., thermophiles (60–80 ${}^{\circ }$C) and hyperthermophiles (>80 ${}^{\circ }$C). The hyperthermophile *Pyrolobus fumarii* shows optimal growth at 106 ${}^{\circ }$C, and can still grow at a maximal temperature of 113 ${}^{\circ }$C [22]. This is truly impressive, and far above the temperatures that typically cause proteins in mesophiles to denaturate.

The external pH surrounding microorganisms is another interesting parameter. In fact, one can find microorganisms favoring all sorts of pH values. Acidophiles grow best in highly acidic environments: *Picrophilus oshimae*—a record holder in terms of acidity—shows optimal growth at pH 0.7 [12]. On the other side, at high pH values, one finds so-called alkaliphiles, e.g., *Natronbacterium gregoryi*, which can still grow at as high as pH 12. Habitats where one finds such extremophiles are e.g., acidic hot springs (acidophiles) and soda lakes (alkaliphiles) [12].

Another highly interesting class of extremophiles is halophiles, which can be subdivided into slight, moderate and extreme halophiles, depending on the [NaCl] which they subsist. Extreme halophiles, which can still grow in even saturated NaCl solutions, can for instance be found in the Dead Sea or in the Great Salt Lake in Utah, USA [14,23].

Further classes of extremophiles include microorganisms that thrive at high external pressures (barophiles), considerable concentrations of heavy metals (metalophiles) or UV and ionizing radiation (radiophiles). Microorganisms that thrive best under more than one extreme environmental factor, are termed polyextremophiles [13]. There are certain combinations of two extreme environmental factors that are frequently found in nature, for instance: (i) high *T* and low pH—occurring in hot springs or volcanic areas; (ii) high [NaCl] and high pH—occurring in soda lakes; (iii) low *T* and high *p*—found in the deep sea; and (iv) high *T* and high *p*—existing in/around deep sea HT vents [12].

The mere existence of *extremophile* microorganisms demonstrates that certain biopolymers (such as proteins) cannot only withstand extreme *T*, *p*, salinity, pH, radiation or presence of heavy metals, but even retain their biological function under these conditions. One question immediately arises: How do extremophiles actually manage to subsist in these extreme conditions or even combinations of extreme conditions? Clearly, the answer to this question is highly complex and specific to each class of extremophiles. For thermophiles for instance, both RNA and DNA have been shown to be stabilized/shielded by complexes of $K^{+}$ and ${\mathrm{Mg}}^{2+}$ with polyamines (which are excellent ligands due to their nitrogen donors) [26]. In general, protein structures in thermophiles must be stabilized with respect to high *T*. This is effectively ensured by increasing the number of weak intermolecular interactions, such as H-bonding, ion pairing and disulfide bridging, hence generating more rigid structures [26,27]. So, what can we learn therefrom for materials synthesis? We can infer that organic compounds, such as proteins, can remain both molecularly and structurally (i.e., with respect to secondary and tertiary structures) intact at e.g., elevated temperatures, if “cleverly” designed. For the example of thermophiles, this “clever” molecular design includes high molecular rigidity through increasing the number of weak intermolecular interactions. As will become clear from the following subsection, the design principle of molecular rigidity extends beyond microorganisms, and is also of major importance for HT vent fauna.

#### 2.1.2. Hydrothermal Vent Fauna

**Figure 2:**
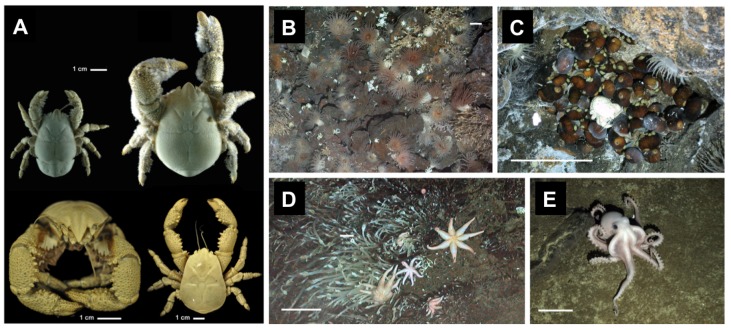
Photographs of animals living in hydrothermal (HT) vent habitats. (**A**) Pictures of yeti crabs *Kiwa tyleri* sp. nov. Top: type material; left female, right male. Bottom: paratype, male, frontal (left) and dorsal view (right). (**B**) Anemone field; (**C**) An undescribed peltospiroid gastropod surrounding single *Kiwa* n. sp. and partially covered by *Lepetodrilus* n. sp.; (**D**) An undescribed seven-arm sea star; (**E**) Unidentified octopus. Scale bars: 10 cm for foreground. Pictures (**B**–**E**) taken at ≈2400–2600 m depth. Photographs published under the Creative Commons Attribution (CC BY) license, and adapted from reference [33] (**A**) and reference [29] (**B**–**E**).

In the earth’s crust, considerable amounts of hot water circulate. Exit points/areas of such hot water streams on land are e.g., furmaroles or geyseres, while submarine outlets are so-called HT vents. With their discovery in 1977 (HT vents at the Galapagos rift), it became clear that numerous animals reside in the surroundings of HT vents [28]. These species do not only live in great depth (for instance, the depth of the Galapagos rift is approximately –2.5 km, hence generating a water pressure of ca. 250 bar) and in proximity to rather high temperatures, but they also live without sunlight [28]. Instead of harvesting energy from sunlight, they derive energy from the bacterial oxidation chemicals in the vent fluids [29]. Interestingly, species found in HT vent areas are only rarely found outside the HT vent habitat, i.e., they are mostly endemic and directly dependent on bacteria inhabiting these areas [30,31]. Examples of animals inhabiting HT vent surroundings (see Figure 2 for photographs) include giant tube worms (Siboglinidae) [28], squat lobsters (*Galatheoidea*) [32], limpets [28,29], yeti crabs (Kiwaidae) [33,34], and alvinocaridid shrimps (Alvinocarididae) [35].

As for extremophile microorganisms, two major questions arise: Which molecular features allow HT vent fauna to subsist at such high pressures and increased temperatures? and What can we therefrom derive for materials synthesis? As for extremophile microorganisms, the mere complexity of living systems forbids an all-encompassing answer. We shall, in the following, briefly discuss only one structural feature of HT vent fauna: their skeleton. The two major skeletal structures in animals are collagen and chitin systems [36]. While collagen systems comprise of associations of collagen with non-collagenous proteins, chitin systems comprise of associations of the polysaccharide chitin with non-collagenous proteins [36]. Note that chitin systems are typically used in exoskeletons, while collagen systems are mostly found in endoskeletons. Both skeletal structures contain a high content of biominerals. The endoskeleton (bone) in humans contains hydroxylapatite (a calcium phosphate), while the exoskeleton in arthropods contains calcium carbonate. Interestingly, most species associated with HT vent habitats are invertebrates [37], many of which have a chitin-based exoskeleton that is often biomineralized [38]. For instance, the giant tube worm bears an exoskeleton composed of several concentric layers of chitin [31]. Another example is crustaceans [38], such as the above mentioned yeti crabs. It has been shown that chitin does indeed show high thermal stability of up to ≈400 ${}^{\circ }$C (determined via thermogravimetric analysis in $N_{2}$ atmosphere) [39]. Moreover, when subjected to hydrothermal conditions, i.e., treated with $H_{2}$O at high *T*, chitin cannot be dissolved up to 200 ${}^{\circ }$C, which is believed to be a direct consequence of its high crystallinity [40]. Again, a design principle that we already encountered for extremophile microorganisms occurs: the structural rigidity—in chitin arising from a highly regular polymer and a considerable sum of intermolecular interactions leading to high crystallinity—yields enhanced stability, withstanding high pressure and elevated temperatures.

Most interestingly, chitin is a polysaccharide, and polysaccharides have been shown to often play vital roles as structure and crystallization directing agents in biomineralization [38]. Biomineralization is the technical term for processes that lead to the formation of minerals, i.e., inorganic, crystalline compounds, in living systems [41]. Examples include the formation of ${\mathrm{CaCO}}_{3}$ shells in mollusks, or of ${\mathrm{SiO}}_{2}$ in diatoms [42]. The terms “structure-directing” and “crystallization-directing” here refer to molecules whose presence plays a key role in directing *where* a biomineral will form, and *which* structure (for instance polymorph) will form in an organism. Polysaccharides have been shown to fulfill these roles in e.g., chitons (Polyplacophora) [43], marine glass sponges (Hexactinellida) [44], or diatoms [45]. As we will see in the Section 2.4 (i.e., *Synthesis of Hybrid Materials by Extreme Biomimetics*), both the high stability and structure-/crystallization-directing ability of chitin can be put to use in the synthesis of novel, intriguing materials. Prior to that, geological materials formation processes will be briefly summarized, with a focus on hydrothermal procedures.

### 2.2. Geological Materials Formation Processes

**Figure 3:**
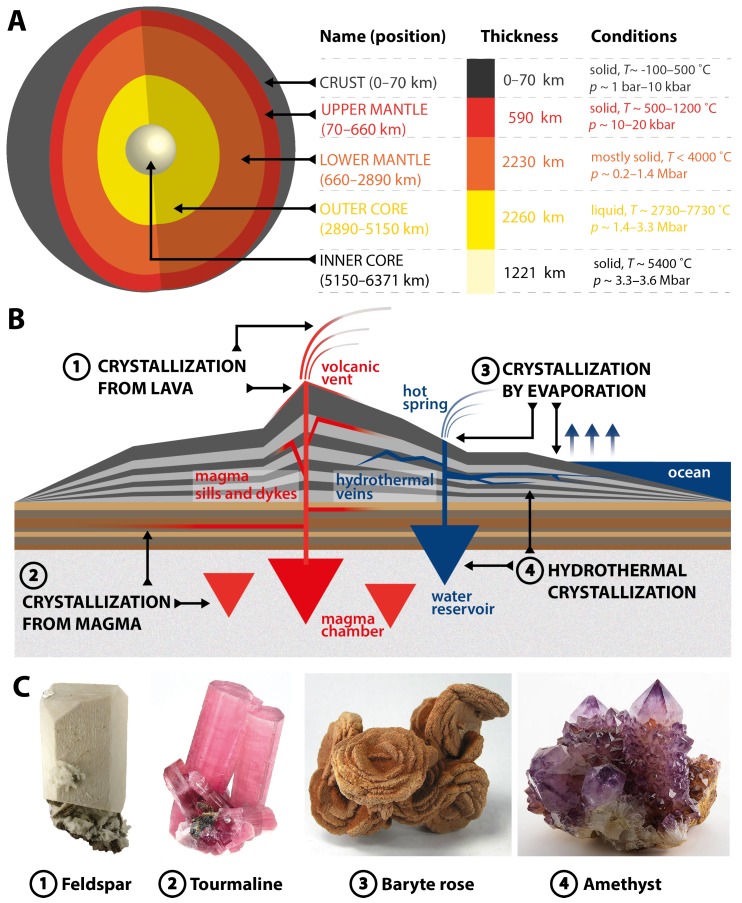
Geological materials formation conditions and processes. (**A**) Structure of the Earth with names, thickness and conditions (p,T) of the different zones. Adapted from [47], and compiled using data from [46]. (**B**) Schematic of different crystallization processes and their loci in the earth’s crust: crystallization from the melt above (1) and underground (2); and from solution above (3) and underground (4). (**C**) Photographs of minerals typically formed by processes (1–4). All photographs published under Creative Commons Attribution (CC BY-SA 3.0) license. Photographs of Feldspar (1), Tourmaline (2) and Baryte rose (3) by Rob Lavinsky, www.irocks.com. Photograph of Amethyst (4) by J. J. Harrison.

In the previous sections, examples of both microorganisms and animals that live—and even thrive—under biologically extreme conditions have been discussed. The extreme habitat of microorganisms living in hot acidic springs or HT vent fauna, arise from geological, volcanic activity. Geological processes that lead to the formation of solid, crystalline materials are typically situated closer to the Earth’s surface than to its center. While physicochemical parameters such as *p* and *T* are—from an anthropocentric point of view—often quite high near the surface, they are not impressive when compared to conditions in the core. In the the Earth’s inner and outer core, temperatures of up to ca. 7800 ${}^{\circ }$C and pressures of up to 3.6 Mbar (=360 GPa) are found [46], see Figure 3A. The Earth’s core consists mainly of Fe and Ni, while crystalline ores start appearing in the mantle [47]. From the mantel on, solid, often crystalline rocks start to appear. In the mantel and crust, much lower *T* and *p* are found, i.e., 500–1200 ${}^{\circ }$C and 10–20 kbar in the upper mantle, and approximately −100–500 ${}^{\circ }$C and 1–10 kbar in the crust (Figure 3A). By the frame of reference of the overall geological activity of the planet Earth, ore formation processes taking place in the Earth’s crust and mantle are not extreme. Mineral—defined as “Any naturally occurring inorganic solid that possesses an orderly crystalline structure and can be represented by a chemical formula” (cf. [47])—formation processes can be, in the simplest approximation, subdivided into four groups (Figure 3B): (i) crystallization from the melt above ground; (ii) crystallization from the melt underground; (iii) crystallization from solution above ground; and (iv) crystallization from solution underground. Other processes such as crystallization from the gas phase shall not be considered herein. Molten rock underground is termed *magma*, and above ground is termed *lava*. When magma chambers, or magmatic fluids in sills or dykes, slowly cool down, certain minerals can crystallize, such as tourmaline (a boron silicate, Figure 3C) or beryl (a beryllium aluminium silicate). Above the ground, lava effluents can give rise to the crystallization of ${\mathrm{SiO}}_{2}$-rich minerals such as feldspar (Figure 3C). Note that the crystallite amount and size depends on the speed of lava cooling [47]. For instance, a rapid volcanic eruption will mainly lead to glassy rocks containing small grains of crystalline minerals. Aside crystallization from the melt, minerals can also originate from aqueous solutions. When such solutions are being narrowed to concentrations above the solubility of the dissolved species, these will crystallize. As such, high concentrations are typically reached by evaporating the solvent $H_{2}$O; minerals originating from this process are typically termed *evaporites*. Evaporites are often found where oceans have retracted. Examples include halite (NaCl) or baryte (${\mathrm{BaSO}}_{4}$) crystals. A prominent example is so-called desert roses, often formed by baryte that slowly crystallizes on sand grains as heterogeneous nuclei (cf. Figure 3C). The fourth mechanism is crystallization from solution below the ground (4 in Figure 3B). Here, reservoirs of ground water—which originate from both $H_{2}$O trickling down from the Earth’s surface (rain, oceans) and the dehydration of rocks by metamorphic activity—are being heated up when in proximity to a hot area such as a magma chamber. In hot $H_{2}$O, the dissolution of ions and atoms from delimiting rocks is enhanced. When the temperature decreases again, the dissolved species reprecipitate and can, at low cooling rates, form rather large crystals. Often, hydrothermal solutions also leach into cracks within rocks, where they form vein deposits upon cooling. Examples of hydrothermally formed minerals are amethyst (Figure 3C), or natural zeolites [8].

In terms of physicochemical conditions, crystallizations from the melt require much higher temperatures (>1000 ${}^{\circ }$C) than crystallizations from solution (hydrothermal systems operate at typically 100–500 ${}^{\circ }$C) [5]. The high temperatures of geological melts can clearly only be reached without degradation by inorganic compounds such as metals, alloys or inorganic minerals. Moreover, in the geological context, solutions are, in the vast majority of cases, aqueous solutions. Clearly, this demands that the precursors of materials that are hydrothermally crystallized are first of all soluble, and second stable, i.e., do not degrade in “hot water”. It appears that the physicochemical properties of water significantly change at increased temperatures. Since these changes in the physicochemistry of water strongly impact the solubility of solutes and chemical reactions, we shall briefly consider them in the following.

**Figure 4:**
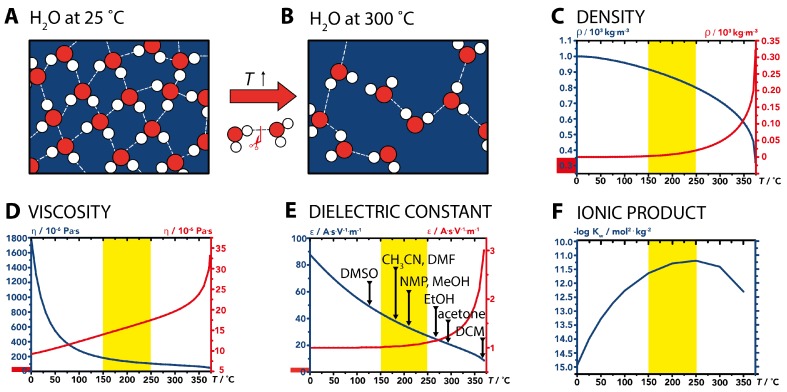
Physicochemical properties of water at elevated temperatures. (**A**,**B**) Schematic of the H-bonding situation in H${}_{2}$O at room temperature (**A**) and at 300 ${}^{\circ }$C (**B**). (**C**–**F**) Evolution of physicochemical properties with *T* for both saturated H${}_{2}$O${}_{\left(l\right)}$ (blue) and H${}_{2}$O${}_{\left(g\right)}$ (red): density ρ (**C**); viscosity η (**D**); static dielectric constant ϵ (**E**) with ϵ of common organic solvents (DMSO: Dimethyl sulfoxide; CH${}_{3}$CN: Acetonitrile; DMF: Dimethyl formamide; NMP: *N*-methyl pyrrolidone; MeOH: Methanol; EtOH: Ethanol; DCM: Dichloromethane) for comparison; and ionic product $K_{w}$ (**F**). The position of the *y*-axis for properties of H${}_{2}$O${}_{\left(g\right)}$ is illustrated by red boxes on the *y*-axis of H${}_{2}$O${}_{\left(l\right)}$. Yellow background emphasizes the *T*-range (150–250 ${}^{\circ }$C) in which most hydrothermal artificial materials syntheses are performed. (**C**–**F**) were plotted using data from references [49,50,51,52].

We all know liquid water as a polar protic medium, which is well suited as a solvent for highly polar compounds such as inorganic ions. However, our picture of water is that of water at ambient conditions. Nevertheless, with increasing temperature (interestingly not so much with pressure), the physicochemical properties of $H_{2}$O change drastically. The changes in properties are a direct consequence of the changes in the H-bonding network of $H_{2}$O, which breaks down with *T*: in fact, the average number of H-bonds per molecule of $H_{2}$O decreases from 3.9 at room temperature (H-bonded network, tetrahedral environment at each O-atom) to 2.4 at 300 ${}^{\circ }$C (loosely/non-connected $H_{2}$O clusters) and saturation pressure (Figure 4A,B) [48].

The changes in H-bonding of $H_{2}$O cause several properties of high-temperature water (HTW) to change significantly as compared to ambient $H_{2}$O. Most important for HTW as a medium for formation reactions and crystallization are the density ρ, the viscosity η, the static dielectric constant ϵ and the ionic product $K_{w}$[53,54,55]. First, both ρ and η rapidly decrease with *T* for $H_{2}$$O_{\left(l\right)}$ (see Figure 4C,D), which strongly affects transport properties: with decreasing ρ and η, the diffusivity in $H_{2}$O increases, which is of importance especially for diffusion-controlled chemical reactions [54]. Note that at “typical” temperatures at which artificial hydrothermal synthesis are carried out (150–250 ${}^{\circ }$C, highlighted by yellow boxes in Figure 4C–F), η($H_{2}$$O_{\left(l\right)}$) is only at $\frac{1}{7}$ of its value at ambient, while ρ($H_{2}$$O_{\left(l\right)}$) has decreased by 20%. Second, the static dielectric constant, ϵ, which reflects the polarity of a solvent, also decreases with *T* (Figure 4E). In Figure 4E, ϵ of common organic solvents at room temperature are indicated. Strikingly, ϵ($H_{2}$$O_{\left(l\right)}$) in the “typical” T-range of hydrothermal systems (orange area), is comparable to polar aprotic solvents such as *N*-methyl pyrrolidone (NMP), acetonitrile (${\mathrm{CH}}_{3}$CN) or dimethylformamide (DMF). This is beneficial for the crystallization of certain minerals from solution. Consider for instance silicates, which form by polycondensation of *ortho*-silicic acid, ${\mathrm{Si}(\mathrm{OH})}_{4}$, in the presence of metal cations. In the lower hydrothermal regime, the increased apolarity favors the dissolution of polysilicic acids over *ortho*-silicic acid [56]. Third and last, the ionic product of water, $K_{w}$, increases with *T*, and has its maximum at 250 ${}^{\circ }$C, i.e., again, in the “typical” hydrothermal regime of approximately 150–250 ${}^{\circ }$C (Figure 4F). Since $K_{w}$ is the product of [${\mathrm{OH}}^{-}$] and [$H_{3}$$O^{+}$], its increase reflects the increasingly dissociated nature of $H_{2}$O with *T* [54]. The fact that $K_{w}$ is three orders of magnitude higher at 250 ${}^{\circ }$C than at room temperature, allows for the reaction medium $H_{2}$O itself to act as an acidic, basic or even powerful acid/base bicatalyst. In fact, it has been shown that the increase in the reaction rate of acid- and base-catalyzed reactions in HTW goes far beyond the expected acceleration due to increased *T* [57]. The increased $K_{w}$ is clearly beneficial for many mineral formation reactions. Let us again consider the example of silicates. The polycondensation of ${\mathrm{Si}(\mathrm{OH})}_{4}$ to polysilicic acids—and also polysilicates in the presence of cations—is catalyzed by both acids and bases [58]. Hence, the increase in both [${\mathrm{OH}}^{-}$] and [$H_{3}$$O^{+}$] is clearly beneficial in the genesis of minerals that form by polycondensation.

These changes in physicochemical properties of $H_{2}$O upon increased *T*, make HTW a highly interesting reaction medium. In the following (cf. Section 2.3), a small number of examples of geomimetic hydrothermal materials syntheses of inorganic compounds will be summarized. The recent discoveries of the possibility to hydrothermally generate crystalline organic materials will be discussed in detail.

### 2.3. Geomimetics for Materials Synthesis

In the previous section on geological materials formation, we have seen that ore genesis can be categorized in solution and melt-based crystallization processes. While numerous melt-based artificial mineral syntheses have been used for over a century (e.g., the Verneuil process for producing ruby) [59], these shall not be further commented on herein. In the context of this article, we will limit our considerations to artificial hydrothermal syntheses of materials. In fact, the physicochemical properties of HTW are not only beneficial for the synthesis of inorganic materials, but also for generating organic materials in nothing but water. Only hydrothermal syntheses leading to crystalline materials shall be termed geomimetic, in analogy to the high crystallinity in natural ores of hydrothermal origin [8].

#### 2.3.1. Geomimetic Hydrothermal Synthesis of Inorganic Materials

**Figure 5:**
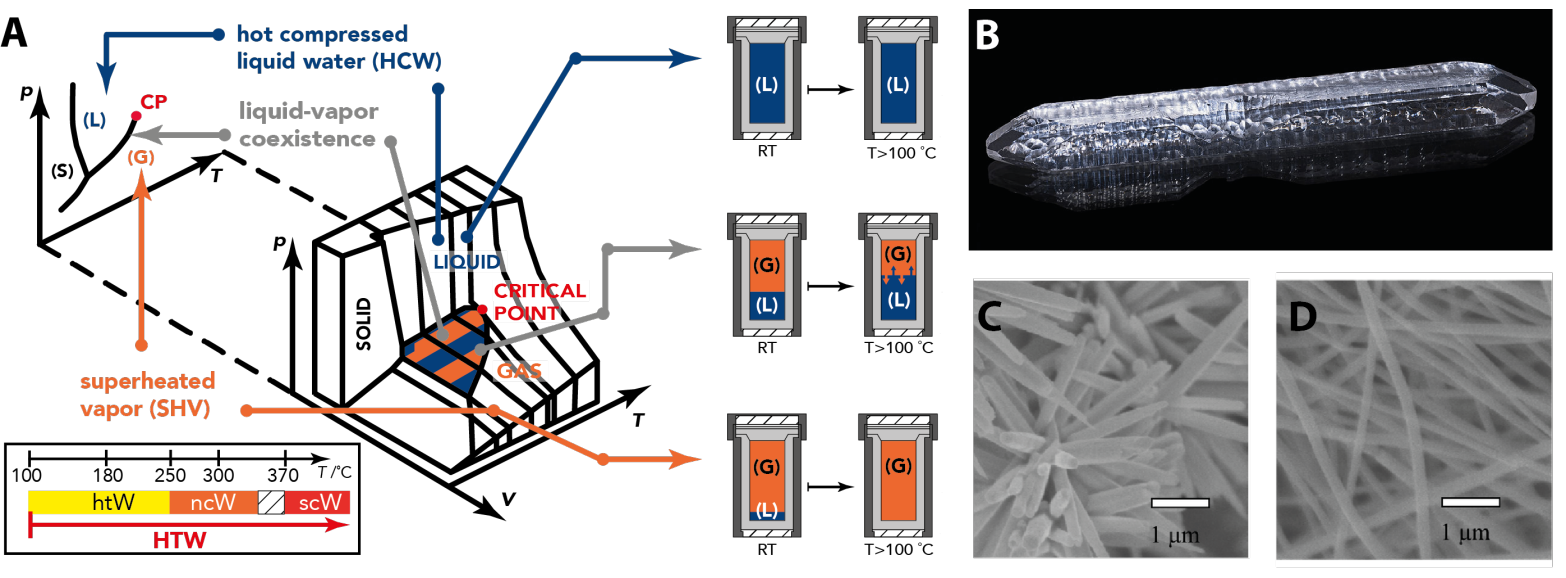
Geomimetic hydrothermal synthesis of inorganic materials. (**A**) pressure–temperature (p,T) and pressure–temperature–volume (p,V,T) phase diagrams of pure H${}_{2}$O with indication of the high-temperature water (HTW) scenarios: superheated vapor (SHV), hot compressed water (HCW), and liquid–vapor coexistence, and the experimental initial autoclave fillings that generate these scenarios (right). *T*-ranges of hydrothermal water (htW), nearcritical water (ncW) and supercritical water (scW) are indicated on the bottom left. RT: Room temperature. (**B**) Photograph of a 19.2 × 2.8 cm hydrothermally synthesized quartz single crystal. Image credit: Didier Descouens, published under CC BY-SA 4.0. (**C**,**D**) Scanning electron micrographs of hydrothermally synthesized ZnO nanostructures; Image adapted from [62], published under CC BY-SA 3.0.

The term ‘hydrothermal synthesis’ is commonly employed for reactions carried out in $H_{2}$O as a reaction medium above its atmospheric boiling point (100 ${}^{\circ }$C) and at autogenous pressure [60]. Historically, one commonly made the distinction between the *hydrothermal* and the *pneumatolytic* regime, where the latter referred to supercritical $H_{2}$O [61]. The term “pneumatolytic” is however not employed anymore, as there are no discontinuities above the critical point or $H_{2}$O [61]. Nowadays, HTW is typically subdivided into three regimes (cf. Figure 5A): (i) hydrothermal water (htW): $H_{2}$O at 100 ${}^{\circ }$C <T< 250 ${}^{\circ }$C; (ii) nearcritical water (ncW): $H_{2}$O at 250 ${}^{\circ }$C <T< 350 ${}^{\circ }$C; and (iii) supercritical water (scW): $H_{2}$O at $T<T_{c}=374$ ${}^{\circ }$C. Note that the fact that the ncW regime does not end directly below $T_{c}$ ($374^{\circ }$C) has practical reasons: in close-proximity to the critical point (CP), even the slightest changes in *T* generate drastic changes in *p*, which makes it extremely hard to operate aqueous systems above 350 ${}^{\circ }$C in a controlled manner. HTW (the overarching term that refers to any aqueous system at T>100 ${}^{\circ }$C, including ncW and scW) is typically obtained by heating a certain amount of $H_{2}$O in a closed steel vessel (a so-called autoclave, Figure 5A, right side), i.e., under isochoric conditions, to the desired *T*. If one heats a small amount of $H_{2}$O to T>100 ${}^{\circ }$C in an autoclave of a comparatively large volume, one generates purely gaseous $H_{2}$O at rather low *p*, so-called “superheated vapor” (SHV, see Figure 5A, bottom right). SHV is however not very interesting in terms of physicochemical properties: the changes in ρ, η and ϵ in $H_{2}$$O_{\left(g\right)}$ with *T* are relatively small (cf. Figure 4C–E). By heating a given volume of liquid $H_{2}$O at ambient to T>100 ${}^{\circ }$C in an autoclave of the same volume (i.e., the autoclave is already entirely filled at room temperature), one generates liquid $H_{2}$O under high pressure, so-called “hot compressed (liquid) water” (HCW, see Figure 5A, top right). If ambient liquid $H_{2}$O in a limited but higher volume (e.g., at 30 *v*% filling of an autoclave’s volume with $H_{2}$$O_{\left(l\right)}$ at room temperature) is heated to T>100 ${}^{\circ }$C, one retains a mixture of high-temperature liquid and gas, i.e., one operates exactly in the liquid–vapor coexistence area (see phase diagram Figure 5A). The corresponding *p* is generated autogenously. Note that, when HTW contains other species—dissolved or not—the pressure is different from the one predicted by pure water’s phase diagram. If the concentration of other species, and the pressure they generate by themselves (e.g., by decomposition) is relatively small, the predicted *p* of pure $H_{2}$O serves nonetheless as a good approximation. Here, $H_{2}$$O_{\left(l\right)}$ constantly evaporates, and $H_{2}$$O_{\left(g\right)}$ constantly condenses. As we have seen for superheated vapor, the physicochemical properties of $H_{2}$$O_{\left(g\right)}$ are not of as much preparative interest as those of $H_{2}$$O_{\left(l\right)}$. Also, the continuous condensation of $H_{2}$$O_{\left(g\right)}$ and continuous evaporation of $H_{2}$$O_{\left(l\right)}$ in the coexistence regime come at a high energetic cost. Therefore, the use of HCW is generally preferable to the liquid–vapor coexistence. However, HCW implies considerable pressures, which in turn demands that the used autoclaves sustain much higher internal pressures.

Numerous inorganic materials can be generated in HTW. Possible types of materials span from compounds that are, when naturally formed, exclusively of hydrothermal origin (e.g., zeolites), to rather exotic examples, e.g., pure metals such as silver [63], gold [64], or copper [65]. A plethora of examples has been collected and reviewed extensively, for instance by Rabenau [61], and Byrappa and Yoshimura [5]. The material classes that are most synthesized hydrothermally include zeolites [66], synthetic gemstones such as sapphire and quartz [67,68], and a variety of inorganic salts such as oxides, carbonates, sulfates, sulfides, or fluorides [61]. For all these materials, the advantages of hydrothermal crystallization include: (i) the precursors are mostly soluble in HTW, even if they are insoluble in $H_{2}$O at room temperature; (ii) the formation of the desired products is often favored in HTW (e.g., when acid/base catalysis is favorable or even required for formation); and (iii) highly crystalline products can be obtained. Inorganic ionic compounds are inherently easy to crystallize. Synthetic hydrothermal crystallizations additionally employ designed cooling protocols, and can be engineered to profit from temperature gradients, as well as the use of seed crystals [5,61]. Consequently, hydrothermal crystallizations can generate both crystalline nanoparticles (see hydrothermally synthesized ZnO nanoparticles, Figure 5C,D) and single crystals of impressive sizes (up to tens of cm, cf. hydrothermally generated quartz single crystal of 19.2 × 2.8 cm, Figure 5B). The most common set-ups to generate hydrothermal conditions artificially are the already mentioned autoclaves, and sealed thick-walled glass tubes [5,61]. Employing steel vessels allows for various adaptions of the set-up, including the use of, e.g., gas inlets, stirrers, or diaphragms for the generation of different concentration zones [61]. In contrast, the use of glass tubes is cheaper but also less sophisticated.

Overall, numerous inorganic materials can be generated via geomimetic, hydrothermal syntheses. Moreover, recently, geomimetic hydrothermal synthesis of organic compounds was reported. This is discussed in the following section.

#### 2.3.2. Geomimetic Hydrothermal Synthesis of Organic Materials

While ionic inorganic compounds have a predisposition for forming crystalline species—i.e., it is actually more difficult to generate an amorphous glass of an ionic solid, than to crystallize it—polymeric organic materials are typically hard to crystallize. Therefore, it is especially important to repeat that the term *geoimimetic* for hydrothermal synthesis shall only be employed if, in analogy to geological hydrothermal mineral formation, crystalline compounds result.

**Figure 6:**
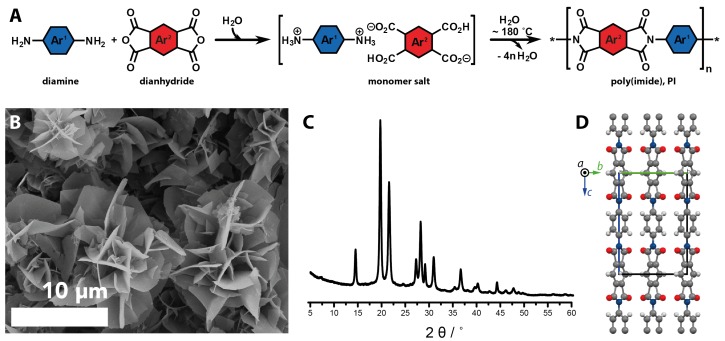
Highly crystalline polyimides by hydrothermal polymerizatiom (HTP). (**A**) Reaction equation of the hydrothermal synthesis of aromatic polyimides (PIs); (**B**) Scanning electron microscopy (SEM) image of poly(*p*-phenylene pyromellitimide) (PPPI); (**C**) Powder X-ray diffraction (PXRD) pattern of PPPI from HTP; (**D**) Crystal structure of PPPI, refined from PXRD data; Shown is the unit cell along *a*, green line = *b*, blue line = *c*, atom color code: carbon = dark grey, hydrogen = light grey, nitrogen = blue, oxygen = red.

In 2003, Chiefari et al. reported that the organic polymers, polyimides, could be synthesized in HTW [69,70,71]. Polyimides (PIs) are condensation polymers, i.e., they are obtained by the elimination of a low-molecular weight byproduct (here $H_{2}$O) upon each monomer addition step. The comonomers generating PIs upon polycondensation are diamines and dianhydrides, see Figure 6A. What becomes immediately clear is the analogy to inorganic polycondensations [8], such as the already discussed generation of silicates by condensation of Si(OH)${}_{4}$ in the presence of cations. Since $H_{2}$O is well suited for being rapidly heated by microwave (MW) irradiation, PI syntheses of short reaction times (down to <1 h) were soon reported in MW-assisted hydrothermal PI synthesis [72,73]. While the latter reports ascertained the feasibility of PI synthesis in HTW at autogenous pressure, little was known about the mechanism. In 2011, the existence of “monomer salt” intermediates was shown [74]. When the comonomers dianhydride and diamine are brought into contact in water at an elevated *T*, the dianhydride first hydrolyzes to the corresponding tetracarboxylic acid and then reacts via acid/base reaction with the diamine to form a monomer salt, see Figure 6A. Such monomer salts can only form in polar protic environments, but not in solvents conventionally used for PI synthesis, i.e., polar aprotic media [75]. Monomer salts have important implications for hydrothermal polymerization (HTP): In the linear PI case, i.e., the polycondensation of an $A_{2}$ with a $B_{2}$ monomer, in all systems reported to date [74,75,76,77,78], monomer salts imparted ‘ideal’ stoichiometry. This 1:1 stoichiometry is a huge benefit in step-growth polymerizations, as these follow Carothers’ law, which states that ‘perfect’ stoichiometries are required to obtain high conversion and high degrees of polymerization ($DP_{n}$). All hydrothermal syntheses of PIs reported until 2014 lead to amorphous PIs. Recently, the HTP of PIs could be broadened by one major finding: under carefully chosen reaction conditions, HTP yields outstandingly crystalline PIs [76]. As a matter of fact, the crystallinity of HT-PIs outmatches the crystallinity of classically produced analoga by far [76,77]. For the obtained high crystallinity, HTP is referred to as a geomimetic technique [8,76]. The first highly crystalline PI synthesized by HTP was poly(p-phenylene pyromellitimide), PPPI [76]. Due to the outstanding crystallinity obtained via HTP, PPPI’s crystal structure (Figure 6D) could be obtained from powder X-ray diffraction (PXRD, cf. Figure 6C), which is extraordinary for an organic, polymeric material [76]. In follow-up work, two further crystalline PIs, poly(*p*-phenylene benzophenone imide) and poly(4,$4^{\prime }$-biphenyl benzophenone imide), could be obtained [77,78], and their crystal structures—to that date still unknown—could also be refined from PXRD data [78]. All three of these polyimides were obtained as fine powders, composed of microparticles of flower-shaped morphology (Figure 6B), which is further indicative of high crystallinity. Moreover, from detailed mechanistic studies employing an extensive set of experiments, several major observations could be made [77,78]: (i) Highly crystalline polyimides are only forming at ≥180 ${}^{\circ }$C in a solution pathway, i.e., from dissolved monomer ions/monomers; (ii) In the lower hydrothermal regime <180 ${}^{\circ }$C, one obtains oligo- and polyimides that are amorphous/semicrystalline at best, also in a solution pathway. This pathway is only observed if the monomer salt is already soluble at these reaction temperatures $T_{R}$. Hence, this pathway can be avoided by using monomer salts that are insoluble at these $T_{R}$, or by rapidly heating to the desired higher $T_{R}\geq 180{\;}^{\circ }$C; (iii) For monomer salts that are soluble below 100 ${}^{\circ }$C, zwitterionic dimers ($H_{3}$$N^{+}$–${\mathrm{Ar}}^{1}$–${\mathrm{NCO}}_{2}$–${\mathrm{Ar}}^{2}$–(${\mathrm{CO}}_{2}$H)${\mathrm{CO}}_{2}^{-}$) could be isolated that form at these $T_{R}$. This result indicates that oligo- and polyimides formed in HTW possess ionic end-groups; (iv) Monomer salts are able to undergo solid-state polycondensation (SSP), if a salt-specific threshold temperature ($T_{P}$) is reached. SSP yields amorphous/semicrystalline products, and happens in an HTP experiment (in dispersion), if $T_{R}\;>\;T_{P}$. SSP can be avoided by either working at very low concentrations, where no nondissolved monomer salt is present, or by using a monomer salt of such a high $T_{P}$ that $T_{P}>T_{R}>180{\;}^{\circ }$C. From the identification of these pathways—and hence also strategies to avoid the unwanted pathways of SSP and polycondensation in the lower hydrothermal regime—the conditions of HTP can be tuned in such a way to obtain highly crystalline PIs. This is a great opportunity to implement HTP for various applications, in which crystallinity in condensation polymers matters.

In fact, several material properties profit greatly from crystallinity. First, thermal and chemical stability are improved in crystalline vs. amorphous materials. In other words, in order to thermally or chemically degrade a certain material, one has to furnish the energy required to break bonds in that material. If the material one wishes to degrade is crystalline, one has to supply the lattice energy in addition to the sum of bond energies. For instance, graphite can be purified by simply “burning away” the less stable amorphous carbon, or amorphous ${\mathrm{SiO}}_{2}$ can be dissolved in alkaline lye, while quartz is only reacting very slowly with hot lye [79]. Second, mechanical performance can be improved by crystallinity. PPPI, which was prepared hydrothermally with outstanding crystallinity, has for instance a predicted crystallite modulus of over 500 GPa [80], one of the highest values ever reported for a polymer [81]. For comparison: the crystallite modulus of poly(*p*-phenylene terephthalamide) commonly known as Kevlar™—an extraordinarily crystalline polymer—is ‘only’ 180–220 GPa. The macroscopic bulk modulus of a polymer is approaching its crystallite modulus with increasing crystallinity [80]. However, high crystallinity can be accompanied by fracture. This can be avoided by designing three-dimensional (3D) networks: graphite for instance is prone to fracture in parallel to the graphitic planes, while diamond is extremely resistant to fracture. Third, crystallinity is highly beneficial for electronic and optoelectronic properties in organic materials. These arise when (i) electron density is delocalized over a certain number of atoms, and (ii) long-range order, i.e., crystallinity, allows for combination into band structures. In conducting polymers, conjugation of π-electron density over a polymer chain is only enabled if the chain is highly regular, and if chains are packed into crystalline order. In small molecules, bulk electronic conduction requires the small molecules to be packed in a crystalline lattice, where neighbors have overlapping orbitals, e.g., through π-stacks.

Most recently, the first example of the purely hydrothermal synthesis of a hybrid material was presented: Leimhofer et al. reported the hydrothermal co-condensation of ${\mathrm{SiO}}_{2}$ and the semialiphatic polyimide poly(hexamethylene pyromellitimide) [82]. Here, the co-condensation profits from the acidity of one of the polyimide precursors that removes the necessity of an additional condensation promotor for the ${\mathrm{SiO}}_{2}$ formation (which would typically be required). Moreover, the crystallinity of the PI component is also obtained in the presence of ${\mathrm{SiO}}_{2}$ and even to some degree when PI and ${\mathrm{SiO}}_{2}$ are covalently bound to each other [82].

To date, HTP has exclusively been employed for polyimides. However, organic reactions of small molecules in HTW, both synthesis-wise and in the context of degradation studies, have been extensively exploited in the past, as comprehensively summarized by, e.g., Savage and coworkers [53,54,55], and Katritzky, Siskin and coworkers [57,83,84]. Whether hydrothermal synthesis of small organic molecules is geomimetic (with respect to obtaining high crystallinity), is often hard to tell. This is particularly due to the fact that, in preparative organic chemistry research, crystallinity of crude products is rarely investigated, which is especially the case when powders are obtained. Instead, organic chemists typically purify crude products by, e.g., recrystallization, and only study crystallinity (typically by single crystal X-ray diffraction), when single crystals of sufficient size are obtained. To the best of the author’s knowledge, there are to date only two examples reporting the hydrothermal synthesis of highly crystalline low-molecular weight organic compounds. In 2002, Poliakoff and coworkers reported the synthesis of several aromatic benzimidazoles in HTW, mainly in the near-supercritical and supercritical regime [85]. Their hydrothermal syntheses lead—upon slow cooling—to single crystals of three different benzimidazoles. Most interestingly, when solving their crystal structures, two of these compounds crystallized in different forms than previously reported. This indicates that geomimetic hydrothermal synthesis of small organic molecules might be a promising means for the discovery of yet unknown polymorphs. Recently, Unterlass and coworkers reported the hydrothermal synthesis of several perylene and naphthalene bisimides [86]. These well-known organic dyes were hydrothermally obtained without the necessity for excess of any of the starting compounds or the need for catalysts, at high purity. Moreover, the crude products were obtained as highly crystalline powders. Note that both benzimidazoles and perylene and naphthalene bisimides are, mechanistically speaking, obtained by condensation reactions with $H_{2}$O as a byproduct. Clearly, as for inorganic condensations, the physicochemical properties of HTW are highly beneficial also in these cases.

Experimentally, HTP and hydrothermal syntheses of organic small molecules are realized with the same set-ups as used for inorganic hydrothermal syntheses, i.e., using steel autoclaves [76,77], or MW reactors designed for operation at high *T* and *p* [78]. Clearly, the decades of research on inorganic hydrothermal syntheses and the related knowledge of reactor design and operation are a huge advantage.

### 2.4. Synthesis of Hybrid Materials by Extreme Biomimetics

As discussed in the last sections, crystalline inorganic, organic and even inorganic–organic hybrid materials can be obtained using geomimetics. Besides the simultaneous co-condensation of ${\mathrm{SiO}}_{2}$ and a polyimide, no other examples of the hydrothermal bottom-up synthesis of hybrid materials exist to date. In recent years, hybrid materials have however been hydrothermally prepared by extreme biomimetic techniques. In these approaches, the inorganic component is typically synthesized in the presence of a biopolymer. These approaches shall only be briefly commented on, as they have recently been excellently summarized in several chapters in the book “*Extreme Biomimetics*” [87].

As commented on previously, both hyperthermophile microorganisms and HT vent fauna exploit the principle of rigidity to enable stability towards elevated temperatures. This principle has been put to use for the extreme biomimetic synthesis of hybrid materials, by performing inorganic hydrothermal condensation of several metal oxides in the presence of the highly rigid biopolymer chitin [88]. Examples include the preparation of chitin/${\mathrm{SiO}}_{2}$ [89], chitin/ZnO [90], chitin/${\mathrm{ZrO}}_{2}$ [91], chitin/${\mathrm{GeO}}_{2}$ [92], chitin/${\mathrm{Fe}}_{2}$$O_{3}$ [93], and most recently chitin/(Ti,Zr)$O_{2}$ [94]. The majority of the latter examples was generated at very mild hydrothermal conditions, specifically at 90 or 120 ${\;}^{\circ }$C. One characteristic shared by the latter examples is that they are nanocomposites between chitin and minerals, inspired by biomineralized chitin exoskeletons. For instance, in the case of chitin/${\mathrm{SiO}}_{2}$, amorphous silica nanoparticles, condensed into siliceous layers covering the chitin surface were obtained [89]. The structural properties of extreme biomimetically prepared chitin/metal oxide hybrid materials deserve a special mention. In these materials, chitin additionally served as a structural template and was effectively infiltrated by the metal oxides. These structures have been qualified as promising candidates for sophisticated applications, such as in biosensors, heavy-metal adsorbants, or biomedical devices [38]. Besides chitin, other biopolymers have been recently employed in the *extreme biomimetic* hydrothermal synthesis of nanocomposites or mesocrystals, namely the spider-silk protein fibroin (in combination with ${\mathrm{Fe}}_{2}$$O_{3}$) [95,96], cellulose (in combination with ${\mathrm{CaCO}}_{3}$, ${\mathrm{Fe}}_{2}$$O_{3}$ and ZnO, respectively) [97,98,99], and spongin in combination with ${\mathrm{Fe}}_{2}$$O_{3}$ and ${\mathrm{TiO}}_{2}$, respectively [100,101].

## 3. Conclusions

Both *geomimetic* and *extreme biomimetic* hydrothermal syntheses allow for the generation of the most intriguing materials in solely hot water. The benefits of these approaches are that, if employed for materials that have previously not been in the focus of preparative hydrothermal synthesis (specifically purely organic and inorganic–organic hybrid materials), radically novel properties can be obtained. This applies to both chitin–metal oxide composites and polyimides of crystallinities that were inaccessible before hydrothermal syntheses was employed to generate them. While there is still enormous “room to play” due to the mere novelty of both approaches, research in these fields benefits greatly from the profound knowledge of hydrothermal synthesis and systems in inorganic chemistry and the geosciences. Moreover, the fact that purely inorganic, purely organic, and inorganic–organic hybrid materials can now be generated hydrothermally, allows an important fraction of the materials space to be covered. In addition, and as underlined by the principles of *green chemistry* and *green engineering*, as well as numerous governmental directives in various countries, syntheses and processes that are harmful to human health and the environment are simply not acceptable anymore. Chemical production companies have already banned numerous harmful syntheses and compounds, and with all due right, there are more to follow. In this respect, hydrothermal techniques are most interesting: There are many compounds that are already industrially synthesized hydrothermally, such as zeolites or large gemstone single crystals. Hence, it is safe to say that the chemical industry is already experienced in carrying out large-scale hydrothermal processes. In the long-term, green approaches will only be successful if they generate materials that are equal or even better than their classically synthesized counterparts. *Geomimetic* and *extreme biomimetic* hydrothermal syntheses bear the realistic promise to meet this requirement.
